# Supplementary material for: Stress promotes RNA G-quadruplex folding in human cells
Source: Nat Commun. 2023 Jan 13;14:205. doi: 10.1038/s41467-023-35811-x (PMC9839774; doi:10.1038/s41467-023-35811-x)
Supplement: Supplementary file 1 — Supplementary Information [file 41467_2023_35811_MOESM1_ESM.pdf]

Supplementary materials for

RNA G-quadruplexes are stress-responsive elements in human cells

Prakash Kharel[1†], Marta Fay[1†], Ekaterina V. Manasova[2], Paul J. Anderson[1,3], Aleksander V. Kurkin[2], Junjie U. Guo[4*], and Pavel Ivanov[1,3*]

[1]Brigham and Women's Hospital, Harvard Medical School, Boston, MA 02115, USA.

[2]Chemistry Department of Lomonosov Moscow State University, 119991, Moscow, Russia.

[3]Harvard Medical School Initiative for RNA Medicine, Boston, MA 02115, USA.

[4]Department of Neuroscience, Yale School of Medicine, New Haven, CT 06520, USA.

[†]PK and MF contributed equally to this work.

*****Correspondence: junjie.guo@yale.edu; Tel.: +1-203-737-4439
pivanov@rics.bwh.harvard.edu; Tel.: +1-617-525-1233

Supplementary Table 1. List of the rG4/ non-rG4 oligos

Name	Sequence (5'-)
Control1	r-UGAAGGGUUUUUGUGUCUCUAUUUCCUUC
Control2	r-GAGUGAGAGAGUGAGAGAGA
Control3	r-GUGAGUGUAGCUCAGUGGUAGAGCGCGUGC
MMP	r-GAGGGAGGGAGGGAGAGGGA
5'tiRNA^{ala}	r-GGGGGUGUAGCUCAGUGGUAGAGCGCGUGC
LMNB1	r-AGGGUGGGGGAGGGAGGUGGAGGGAGGGAAGGGU
APP	r-CGGGGCGGGUGGGGAGGGGU
APH1A	r-UGGGGUGUGGGAGGGGCGGGG
HIRA	r-GGGGGAAUGGGGCAAAGGGGUGGGCGA
CDKN1B	r-GGGGAAGGGAGGGCAGGGTGGGGC

Supplementary Table 2. List of the primers

Name	Sequence (5'-)
BACTN F	GGAAATCGTGCGTGACATTAAG
BACTN R	TAGTCCGCCTAGAAGCATTTG
APH1A F	GATGTCCTCGCCCTGTATTT
APH1A R	CCCAAGGAGGTTAGAAGAGTTC
APP F	GAGGTACCCACTGATGGTAATG
APP R	AGCGGTAGGGAATCACAAAG
LMNB1 F	CTTCTTCCCGTGTGACAGTATC
LMNB1 R	CTTCCTCCTCCTCCTCTTCTT
HIRA F	GACTTCTCCACGGCATTCTT
HIRA R	CCACCACTGTCACTTCATTCT
CDKN1B F	GTCAAACGTGCGAGTGTCTA
CDKN1BR	TGCAGGTCGCTTCCTTATTC
28S rRNA F	CGGGATAAGGATTGGCTCTAAG
28S rRNA R	CTGTGGTTTCGCTGGATAGT
18S rRNA F	ACGTCTGCCCTATCAACTTTC
18S rRNA R	CCGCGGTCCTATTCCATTATT

Increased rG4 formation under different stresses in U2OS and COS7 cells

RNA G-quadruplex formation was probed using starvation, cold shock, and heat shock stresses in U2OS cells and COS7 cells. Cold shock (10 °C overnight) and starvation stress affect rG4 formation similarly in both cell types. Both cell types showed slightly enhanced BG4 signals with heat shock (42 °C for 30 min).

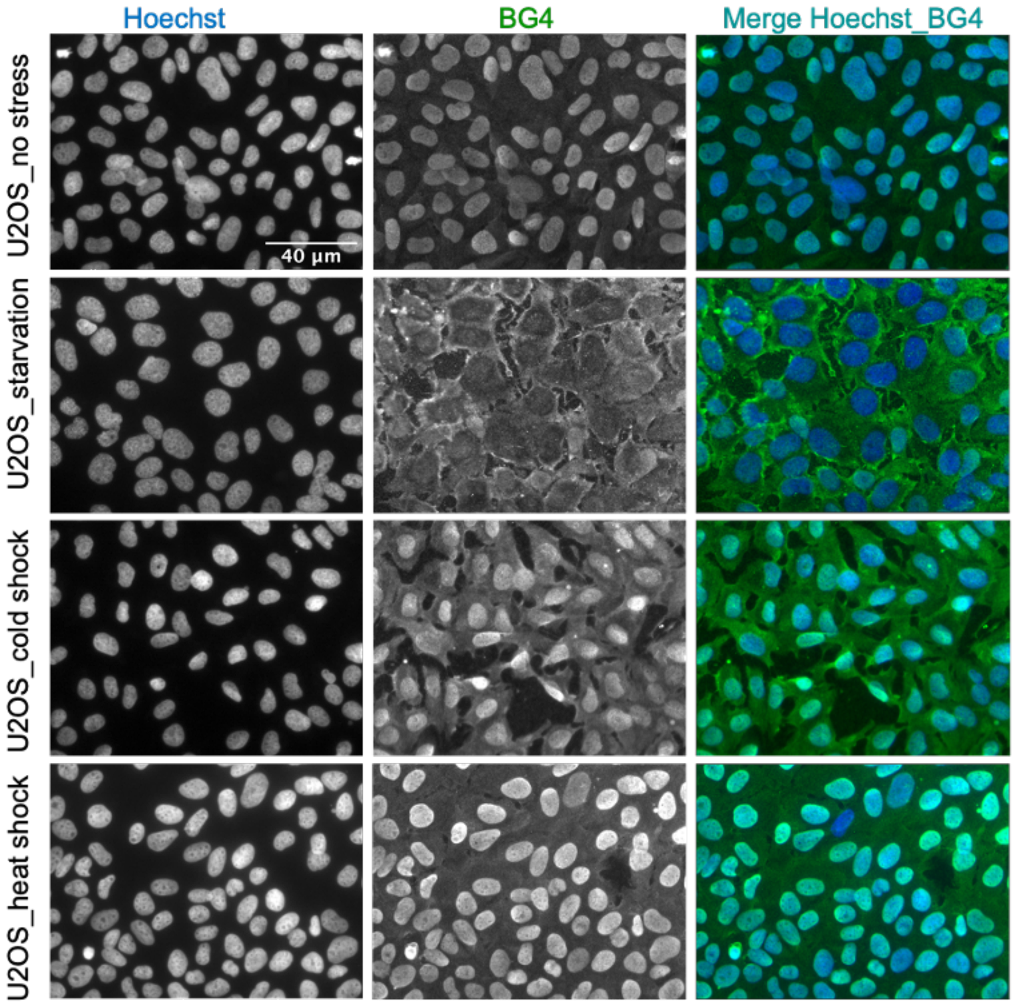

Supplementary Figure 1. RNA G-quadruplex folding is enhanced in U2OS cells under different stresses. From the top, no stress, starvation, cold shock, and heat shock, respectively.

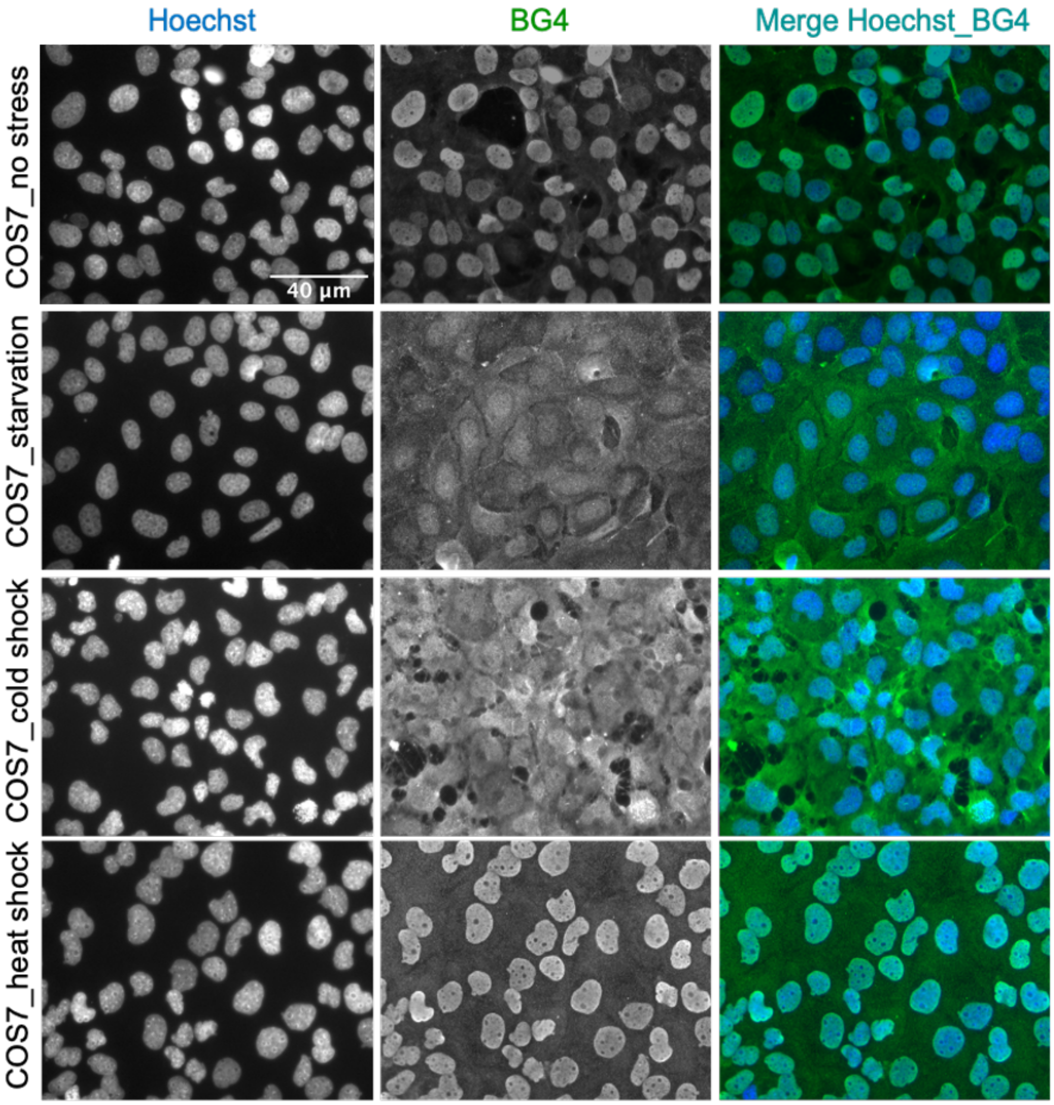

Supplementary Figure 2. RNA G-quadruplex folding is enhanced in COS7 cells under different stresses. From the top, no stress, starvation, cold shock, and heat shock, respectively.

Peroxidase-like activity of rG4-hemin complexes

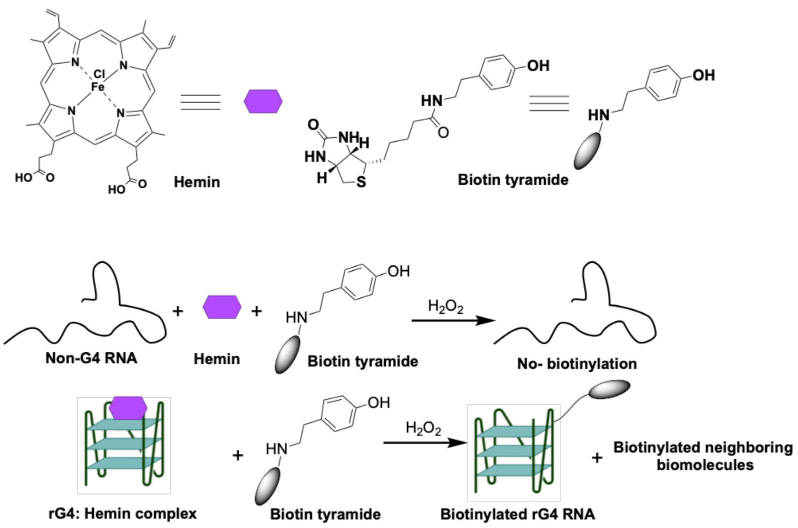

RNA	*5'tiRNA^{ala}	APP rG4	G4C2 rG4	JAK2 rG4	LMNB1 rG4	VEGF rG4	Pre biotinylated 5'tiRNA^{ala}	Non-rG4 1	Non-rG4 2	APP rG4
Hemin	+	+	+	+	+	+	-	+	+	-
Biotin tyramide	+	+	+	+	+	+	-	+	+	-
H_2O_2	+	+	+	+	+	+	-	+	+	-
SA	+	+	+	+	+	+	+	+	+	+

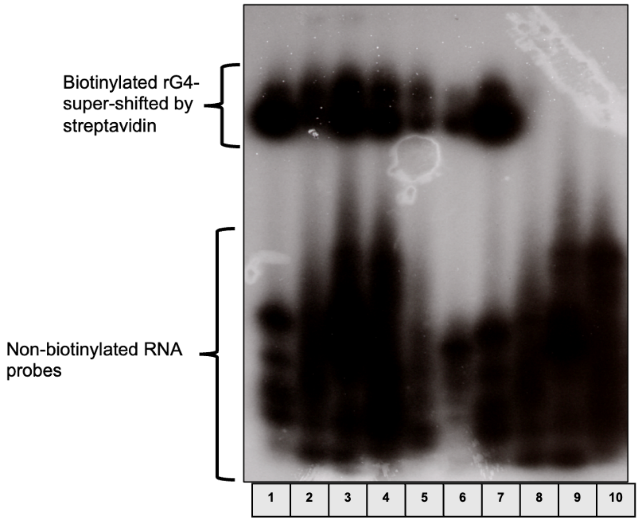

Supplementary Figure 3. rG4 biotinylation scheme. When rG4s are incubated with hemin. and sensitized with H_2O_2, rG4s can be biotinylated in the presence of a suitable substrate (e.g., biotin-tyramide). On the right, the gel shift assay showing a super-shift of biotinylated rG4s in presence of streptavidin used for the biotin detection (Lanes 1-6); super-shift of a positive control (pre-biotinylated oligo), no shift of control RNAs (Lanes 8-9), and no shift of rG4 under non-biotinylating environment.

Circular dichroism (CD) characterization of rG4 formation by DMSeq-identified mRNA-derived oligos representing putative rG4 regions

JASCO J815 spectropolarimeter was used to collect CD spectra. The oligonucleotides were dissolved in 150 mM K+ in $T_{10}E_{0.1}$ buffer. Quartz cuvettes (a 1 mm path length) were used with sample volumes of 200 µL to achieve a sample concentration of 5 µM. Spectra were collected in the range between 220 and 320 nm at 20 °C from three scans, and a buffer baseline was subtracted from each spectrum. CD was expressed as the difference in the molar absorption of the right-handed and left-handed circularly polarized light. An increased peak intensity of the oligo under K^+ environment at 260-265 nm and a trough at 240 nm, which shows a reduced peak intensity under Li^+ environment suggests the formation of a G4. All the selected candidates show the G4 characters in CD while the control oligo that cannot form a G4 structure shows no change in the CD behavior.

Electromobility gel shift assay and NMM staining of the candidate oligos

40 pmoles of candidate oligos (folded as above) were run in 20% non-denaturing gel electrophoresis and stained with NMM and SYBR Gold separately.

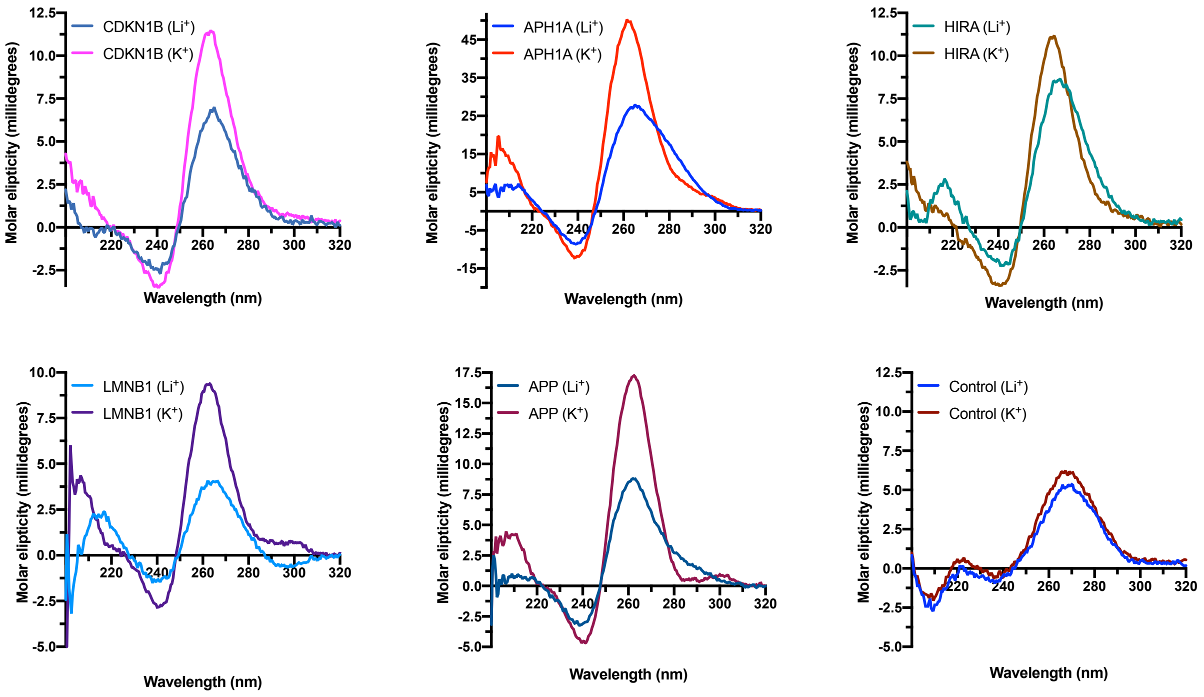

Supplementary Figure 4. CD spectra of candidate oligos show rG4 features which are absent in the control oligo.

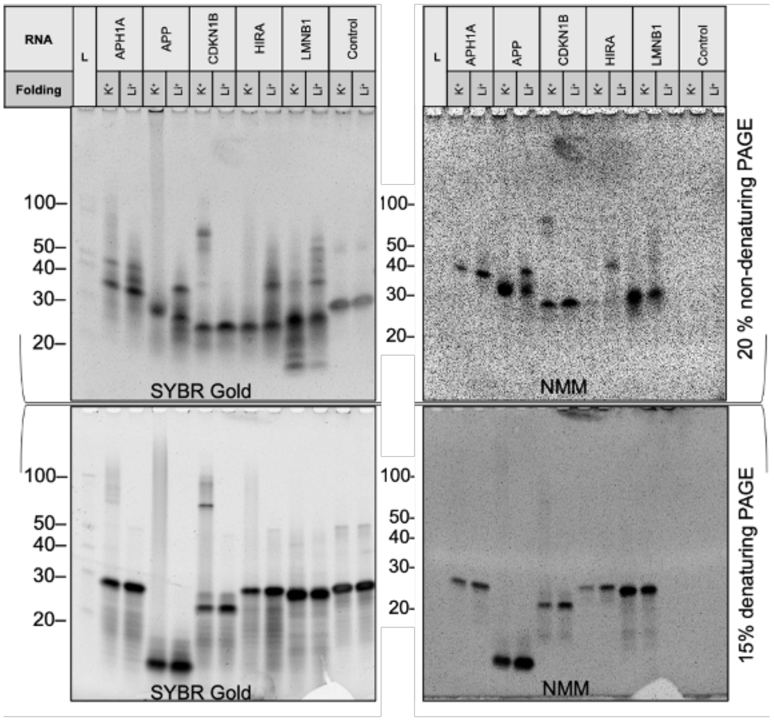

Supplementary Figure 5. Electromobility gel shift assay coupled with G4-specific dye (N-methyl Mesoporphyrin IX, NMM) staining indicates the formation of rG4s by the candidate oligos even under experimentally harsh conditions (Li^+ and denaturing PAGE). SYBR Gold detects nucleic acids non-

selectively while NMM selectively binds to parallel G4s. The ladders represent RNA size markers with molecular weight in Dalton.

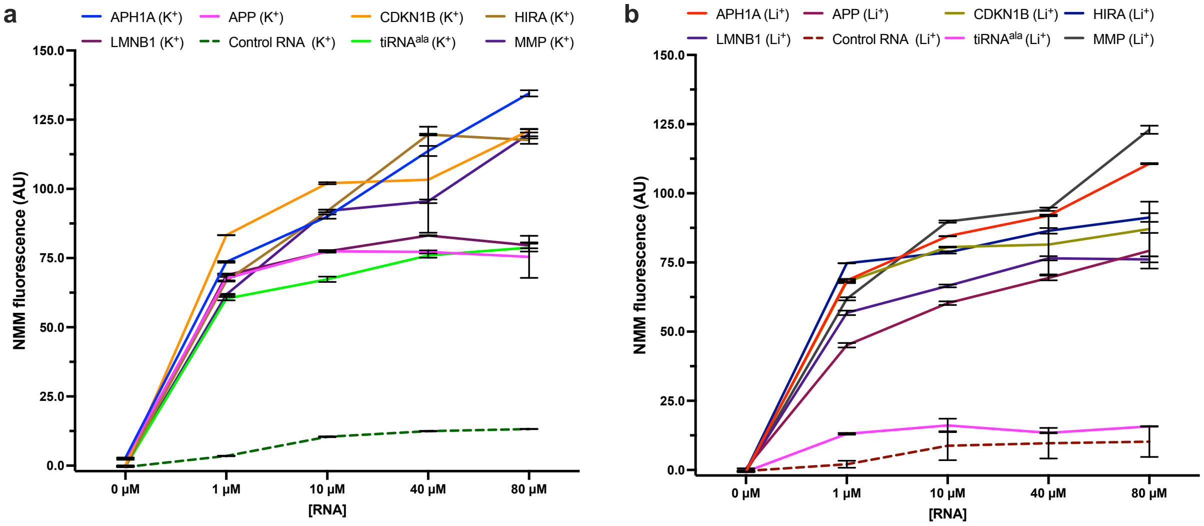

Supplementary Figure 6. NMM fluorescence assay further confirms the formation of rG4s by candidate oligos (both in K^+ and Li^+ conditions). Control RNA is a non-rG4 oligo, and 5'tiRNA^{ala} and MMP are positive controls (as it has been previously shown that 5'tiRNA^{ala} mostly stays in the non-rG4 form under Li^+ environment[1] while MMP can stay in rG4 form even under Li^+ environment[2]). **a.** NMM fluorescence under K+ environment, Data are presented as mean values +/- SD, n=3 and **b.** NMM fluorescence under Li+ environment, Data are presented as mean values +/- SD, n=3.

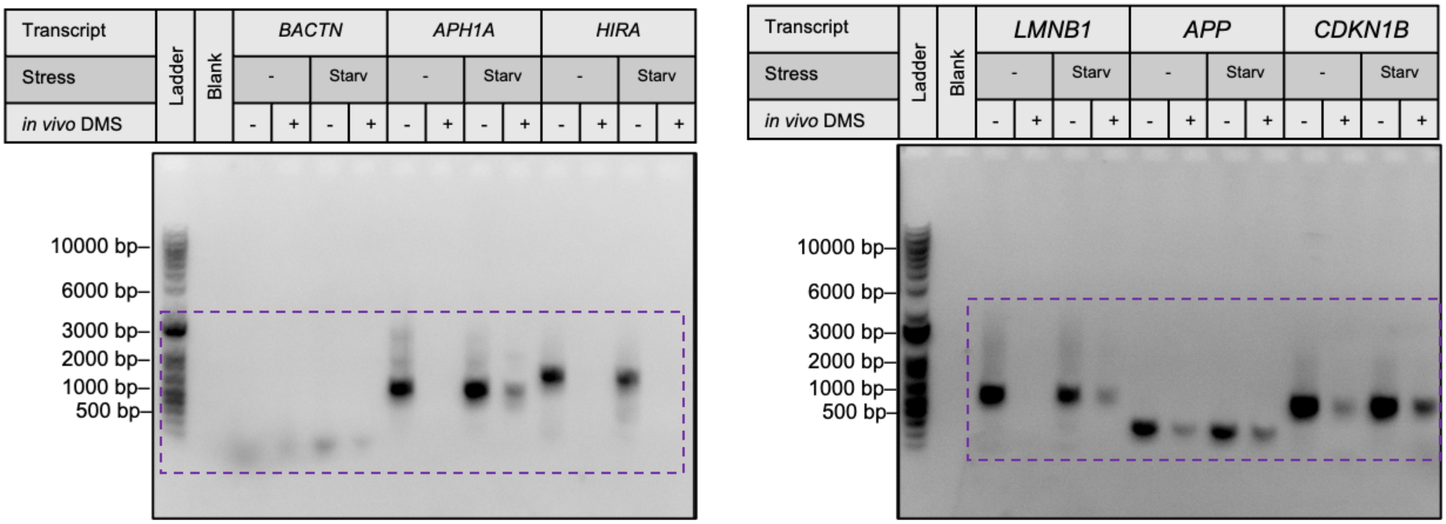

Supplementary Figure 7. Source image for Fig. 3.i. The purple boxes indicate the areas cropped to demonstrate the results in Fig. 3.i. The ladder represents DNA base pair size markers with molecular weight in Dalton.

Stability of control (non-rG4) RNAs under stress

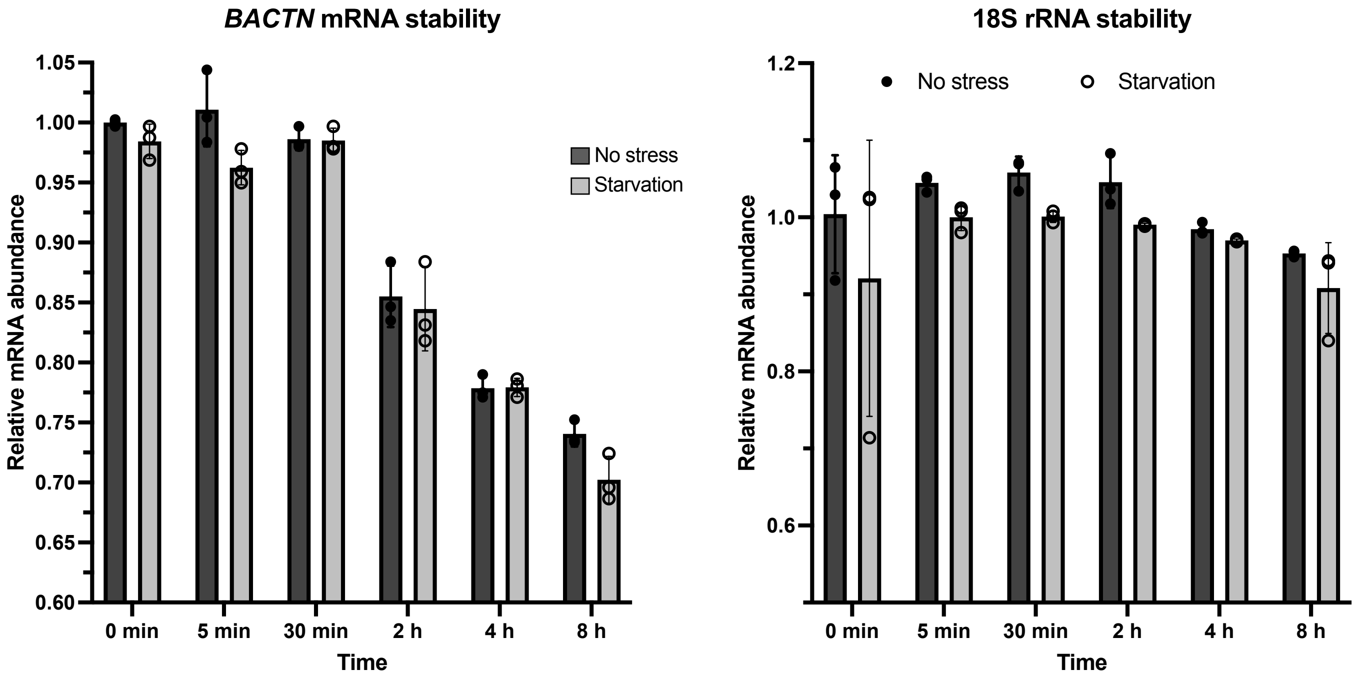

Supplementary Figure 8. Stability of control RNAs over time. There is no significant difference in the stability of non-rG4 RNAs (BACTN and 18S rRNA) under no-stress vs starvation environment. Data are presented as mean values +/- SD of different transcripts at different time points, n=3. Multiple paired t-tests.

Reversible nature of stress-induced rG4 folding

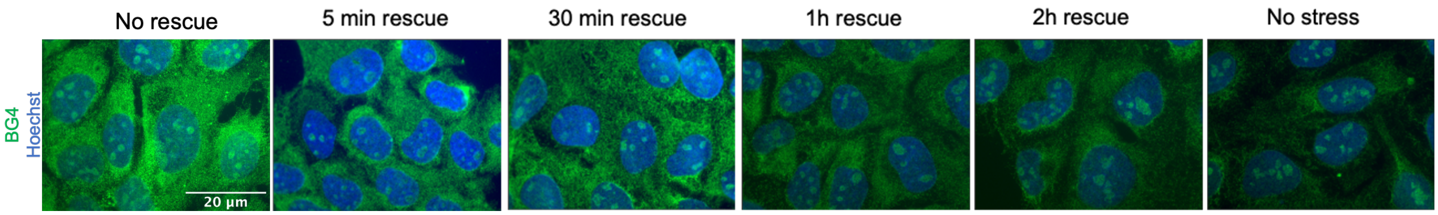

Supplementary Figure 9. Representative images illustrating the kinetics and the reversibility of starvation-induced rG4 folding upon the rescue of the stress (Quantification of relative BG4 fluorescence is presented in **Fig. 4c**).

Synthesis and characterization of biotinylated NMM

Common reagents and solvents were purchased from commercial suppliers and used without further purification unless otherwise stated. Tetrahydrofuran (THF) was distilled from sodium-benzophenone under an argon atmosphere. Reaction progress was monitored using analytical thin-layer chromatography (TLC) on pre-coated silica gel GF254 plates (Macherey Nagel GmbH & Co. KG, Düren, Germany), and spots were detected under UV light (254 and 366 nm). Compounds were purified with flash column chromatography with a silica gel and particle size of 40–63 μM (Merck, Darmstadt, Germany) as the stationary phase and hexane/ethyl acetate or dichloromethane/methanol mixtures as eluent systems. Nuclear magnetic resonance spectra were measured on a Bruker AV-400 NMR instrument (Bruker, Karlsruhe, Germany) in deuterated solvents (DMSO-d6 or CDCl3). Chemical shifts are expressed in ppm relative to DMSO-d6 or CDCl3 (2.50/7.26 for 1H; 39.52/77.16 for 13C). The following abbreviations are used to set multiplicities: s = singlet, d = doublet, t = triplet, q = quartet, m

= multiplet, br. = broad. Measurements for verification and purity of the compounds were performed by LC/MS. LC–MS/MS data were obtained using a Dionex Ultimate 3000 liquid chromatograph (Dionex, USA) connected to an AB Sciex Qtrap 3200 mass spectrometer (AB Sciex, Canada). LC separation was carried out on a Shim-pack GIST C18-AQ (150 mm × 2.1 mm, 3 μm, Shimadzu, Japan) column. Mobile phase consisted of the mixture of 0.1% (v/v) formic acid in water (A) and acetonitrile (B). Separation was performed in isocratic mode 10% : 90% (A : B). The mobile phase flow rate was 0.3 mL/min. The injection volume was 10 μL. Compounds were detected at λ = 254 nm. All high-resolution mass spectra (HRMS) were measured on AB Sciex TripleTOF 5600+ instrument equipped with DuoSpray (ESI) ion source. Samples were directly injected in the ion source in acetonitrile or methanol solutions acidified by formic acid. The melting points were measured in open capillaries and presented without correction. Synthetic details are available in supplementary materials.

Synthesis of Biotin-linker (1)

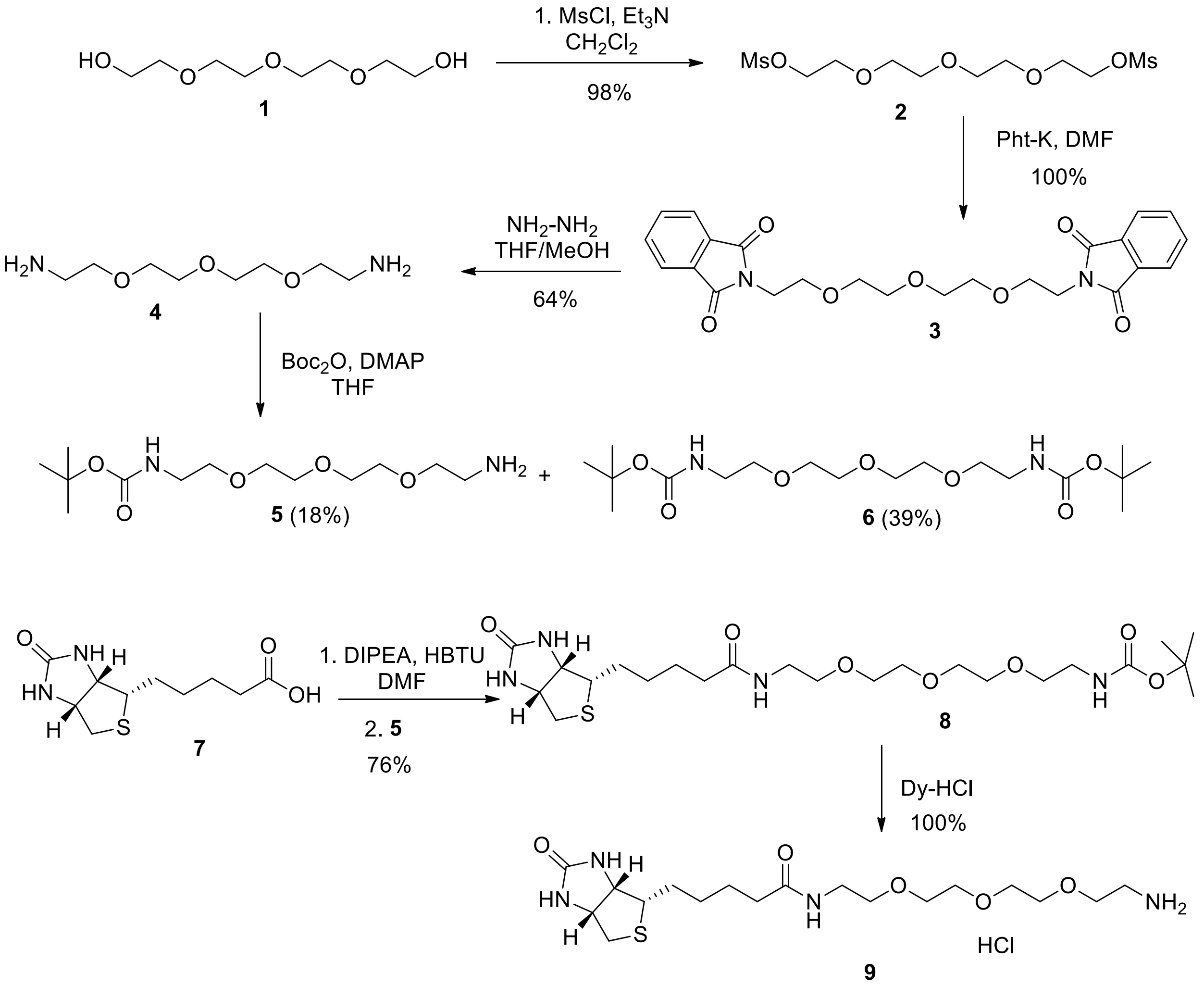

Tetraethylene glycol dimethanesulfonate (2)

Methanesulfonyl chloride (6 mL, 76.82 mmol) was added dropwise to a stirred solution of PEG4-di-OH **1** (4.455g, 22.59 mmol) and TEA (15 mL, 107.08 mmol) in dichloromethane (20 mL) at 0 ^{0}C. After the addition was complete, the resulting mixture was stirred at room temperature for one day. Water was added to quench the reaction. The organic phase was separated, and the aqueous phase was extracted with dichloromethane (2 x 50 mL). The combined organic layers were washed with brine (3 x 60 mL), dried over Na_2SO_4, filtered, and the solvent removed under reduced pressure to afford the desired product. Yield 7.75 g (98%).
^{1}H NMR ($CDCl_3$, 400 MHz) δ: 4.37-4.34 (m, 4 H), 3.76-3.73 (m, 4 H), 3.67 -3.60 (m, 8 H), 3.05 (s, 6 H).

1,11-Diphthalimido-3,6,9-trioxaundecane (3)

A suspension of 3.03 g (8.65 mmol) **2** and 3.85 g (20.77 mmol) potassium phthalimide in 100 mL abs. DMF was heated for16 h at 80°C. The solvent was removed in vacuum and the residue was suspended in 150 mL DCM and filtrated. The filtrate was washed with water (3 x 100 mL), dried over Na_2SO_4, filtrated and concentrated in vacuum. Yield 3.93 g (100%).
^{1}H NMR ($CDCl_3$, 400 MHz) δ: δ = 7.83 (m, 4H, 2 x 5H,6H-Isoindol), 7.69 (m, 4H, 2 x 4H,7H-Isoindol), 3.86 (t, 4H, *J* = 5.7 Hz, 2 x -CH2-CH2-NPht), 3.69 (t, 4H, *J* = 5.7 Hz, 2 x -CH2-CH2-NPth), 3.54 (m, 8H, PhtN-EtO-[(CH2)2-O-]2-Et-NPhth).

1,11-Diamino-3,6,9-trioxaundecane (4)

Product 3 (3.0 g, 6.6 mmol) was dissolved in anhydrous THF–MeOH (1 : 1 (v/v)). To the THF–MeOH solution, 4.0 mL of hydrazine monohydrate was added and the resultant mixture was stirred at room temperature. After 12 h, the white precipitate was removed by filtration. This filtrating process was repeated at least three times to remove the precipitate. The filtrate was concentrated under reduced pressure to give **4** as a yellow oil. Yield 0.815 g (164%) (815 mg, 64%).

^{1}H NMR ($CDCl_3$, 400 MHz) δ: 3.61–3.66 (m, 16H).

Boc-amine-11-Amino-3,6,9-trioxaundecane (5)

The diamine **4** (3.83 g, 0.02 mol, 1 equiv) in DCM solution (10 mL / mmol) was treated with Boc_2O in default (4.80 g, 0.022 mol, 1.1 equiv) and DMAP (0,024g, 0,2 mmol, 0.01 equiv) for 0.5h at 0 °C. The reaction was stirred at room temperature for 12h. The solvent was removed under reduced pressure. The crude product was purified by silica gel chromatography (EtOAc: hexane 1:1) to yield **5** as a colorless oil (1.08 g, 18% yield) and **6** as a colorless oil (3.04 g, 39% yield).

^{1}H NMR ($CDCl_3$, 400 MHz) δ: 5.27 (s, 1H), 3.65 (m, 12H), 3.52 (q, 3J = 5.9 Hz, 2H), 3.31 (q, 3J = 6.8 Hz, 2H), 2.87 (t, 3J = 5.2 Hz, 2H), 1.45 (s, 9H)

N-Boc-N'-biotinyl-3,6,9-trioxaundecane-1,11-diamine (8)[3]

Biotin **7** (0.731 g, 2.99 mmol, Sigma, 1.0 equiv) was weighed directly into the reaction flask. This was dissolved in anhydrous DMF (25 mL). HBTU (98%, 0.18 g, 9.00 mmol, 1.1 equiv) was added next. The reaction flask was placed under an argon atmosphere and was stirred for 2 h at room temperature. To this solution was added a solution of **5** (1.05 g, 3.591 mmol, 1.2 equiv) and DIPEA (98%, 0.467 g, 0.61 mL, 0.528 mmol, 1.2 equiv) in anhydrous DMF (10 mL) and the solution mixture was stirred overnight at room temperature under argon to ensure a complete reaction. The reaction mixture was then evaporated and subjected to silica gel chromatography in 9:1 $CHCl_3$/MeOH as eluent to afford **6** (1.18 g, 76% yield) as a white solid.

^{1}H NMR ($CDCl_3$, 400 MHz) δ: 6.92 (br t, 1H,CH_2NHCO), 6.82 (s, 1H, NHbiotin), 6.22 (s, 1H, NH_{biotin}), 5.15 (br t, 1H, $NH_{carbamate}$), 4.33 (m, 1 H, CHN H_{biotin}), 4.14 (m, 1H, CHN H_{biotin}), 3.35 - 3.47 (m, 12H, $(CH_2–O)_6$), 3.26 (br q, 2H, J = 4.7, 9.5 Hz, CHS), 3.14 (app q, 2H, J = 5.0 Hz, CH_2NHCO), 2.98 (br q, 1H, J = 4.5 Hz, CHS), 2.73 (dd, 1H, J = 5.0,13.2 Hz, CHS), 2.58 (d, 1H,J = 12.7 Hz, CHS), 2.06 (t, 2H, J = 7.4 Hz, CH_2CONH), 1.51 (m, 4H, $(CH_2)_{2biotin}$), 1.27 (br s, 11H, $(CH_3)_3$,$CH_{2biotin}$).

N-Biotinyl-3,6,9-trioxaundecane-1,11-diamine (9)

To a warm solution of compound **8** (0.655 g, 1.26 mmol) in dioxane (4 ml) was added a 4M solution of HCl in dioxane (2 mL). The mixture was stirred at 50 °C for 0.5 h while oil dropped out in the mixture. TLC (10% MeOH in CH_2Cl_2) indicated the reaction was complete. The oil was dissolved in methanol and the resulting solution was evaporated, the residue was co-evaporated several times with $CHCl_3$, Et_2O and CH_2Cl_2. Yield 0.574 g (100%).

^{1}H NMR (DMSO-d_6, 400 MHz) δ: 7.98 - 8.26 (m, 3H), 7.83 - 7.98 (m, 1H), 4.24 - 4.39 (m, 1H), 4.06 - 4.19 (m, 1H), 3.61 (t, J=5.2 Hz, 2H), 3.53 (d, J=16.3 Hz, 8H), 3.32 - 3.45 (m, 2H), 3.13 - 3.24 (m, 2H), 3.05 - 3.13 (m, 1H), 2.87 - 3.01 (m, 2H), 2.82 (dd, J=12.4, 4.98 Hz, 1H), 2.58 (d, J=12.5 Hz, 1H), 2.07 (t, J=7.3 Hz, 2H), 1.56 - 1.69 (m, 1H), 1.39 - 1.55 (m, 3H), 1.16 - 1.38 (m, 2H).

Synthesis of NMM-IX

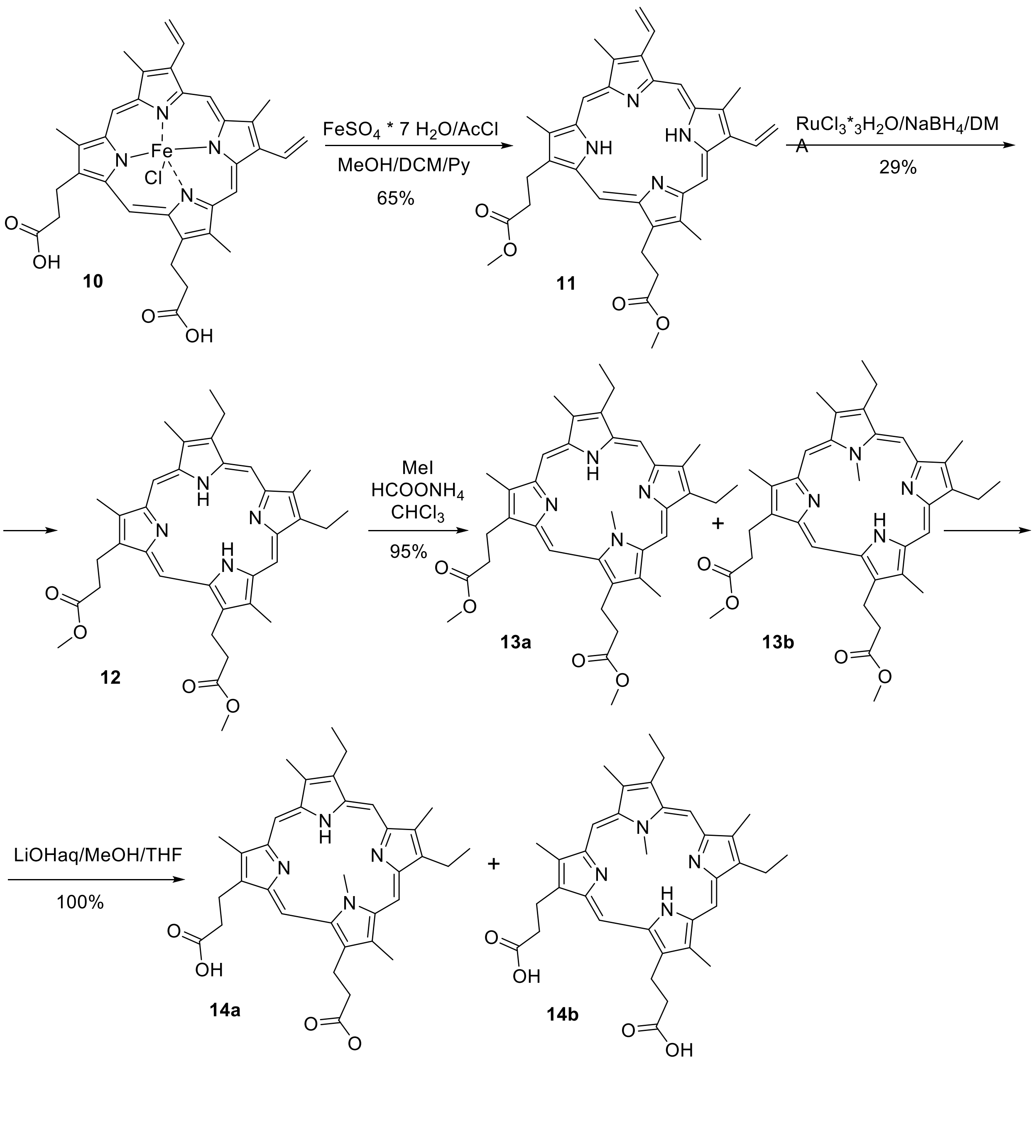

Protoporphyrin IX dimethyl ester (11)[4]

Hemin (**10**) (0.5 g, 0.76 mmol) and pyridine (0.5 mL) were placed in a three-necked flask, then MeOH (30 ml), CH_2Cl_2 (30 ml) and Mohr's salt (1.42 g, 5.1 mmol) were added. Acetylchloride (15 ml, 21.0 mmol) was gradually added under stirring and cooling, while the temperature was kept below 35 °C. The mixture was stirred for 1 h and then diluted with H_2O (50 ml). The bottom organic layer was separated, washed with aqueous ammonia (25%, 30 ml), then with H_2O (20 ml) and dried over anhydrous Na_2SO_4. The product was purified by chromatography on silica gel 60 mesh using CH_2Cl_2 as the eluent. M.p.: 238 - 240 °C. Yield 0.29 g (65%).

^{1}H NMR ($CDCl_3$, 400 MHz) δ: 9.67 - 9.98 (m, 4 H), 7.99 - 8.25 (m, 2 H), 6.29 (dd, J=17.6, 12.32 Hz, 2 H), 6.14 (t, J=10.3 Hz, 2 H), 4.22 - 4.39 (m, 4 H), 3.69 (s, 6 H), 3.40 - 3.60 (m, 12 H), 3.13 - 3.32 (m, 4 H)

Mesoporphyrin IX dimethyl ester (12)[5]

A 100 mL three-neck-round-bottom flask was fitted with a reflux condenser and an oil-bubbler was charged with a solution of **11** (0.591 g, 1.00 mmol, 1 equiv) in DMA (20 mL, 0.05M), $RuCl_3·3H_2O$ (0.131 g, 0.50 mmol, 0.5 equiv), then to this mixture $NaBH_4$ (0.393 g, 10.40 mmol, 10.40 equiv) was added with portions for three times in less than 30 min under N_2 atmosphere and then stirred at 25 °C. After 30 min, the UV–vis spectrum of a sample aliquot showed complete conversion. The mixture was flushed with Ar for 5 min, opened to the atmosphere, and concentrated to 5 mL. The result mixture was transformed into 200 mL water and extracted with 300 mL CH_2Cl_2. The organic phase was collected and washed for three times with water to remove DMA and Ru salts. The crude product was purified by column chromatography (60-80 mesh silica gel). The filtrate was collected and evaporated to dryness to afford **12** as brownish, red, or black solids. M.p.: 211 - 213 °C. Yield: 0.25 g (42%).
^1H NMR (400 MHz, $CDCl_3$) *δ*: 10.21 (s, 1H, 20-H), 10.10 (s, 2H, 5-, 15-H), 9.93 (s, 1H, 10-H), 4.29 - 4.58 (m, 4H, 13-,17-*β*-CH2, *α* to carbonyl), 4.12 (q, J_{HH}=7.6 Hz, 4H, 3-, 8-CH_2CH_3), 3.54 – 3.82 (м, 18H, 2-, 7-, 12-,18-CH_3 and 13-, 17-OCH_3), 3.29 (t, J_{HH}=7.5 Hz, 4H, 13-, 17-*α*-CH2, *α* to carbonyl), 1.89 (t, J_{HH}=7.6 Hz, 6H, 3-, 8-CH2CH3), 3.71 (s, 2H, NH).

N-Methylmesoporphyrin IX dimethyl ester (13)[6]

Mesoporphyrin IX dimethyl ester **12** (0.242 g, 0.407 mmol, 1.0 equiv) was dissolved in $CHCl_3$, (20 mL, 0.02M) and MeI (3.9 mL, 8.886 g, 62.6 mmol, 154.0 equiv) and excess ammonium formate (0.256 g, 4.065 mmol, 10.0 equiv) was added. The mixture was boiled under reflux for 12 hr. cooled and the inorganic salts removed by filtration. The filtrate was concentrated and purified by column chromatography (60-80 mesh silica gel). MeOH-DCM (1:10) eluted a red-brown fraction which on work up and crystallization from $CHCl_3$-MeOH afforded N-Methylmesoporphyrin IX dimethyl ester (**13**) as the mixture of two products (**13a** and **13b**) as purple plates. M.p.: 245 - 250 °C. Yield: 0.23 g (95%). R_f = 0.2 (CH_2Cl_2/MeOH 20:1). **13a**. rt = 1.91 min. LC-MS: m/z [M+H]+ = 609 Da, **13b**. rt = 1.98 min. LC-MS: m/z [M+H]+ = 609 Da.
^1H NMR (400 MHz, $CDCl_3$) *δ*: 10.34 - 11.01 (m, 4 H), 3.74 - 4.80 (m, 15 H), 3.50 - 3.73 (m, 13 H), 3.27 - 3.50 (m, 4 H), 2.97 - 3.08 (m, 1 H), 1.92 - 2.02 (m, 3 H), 1.88 (t, *J*=7.6 Hz, 1H), 1.50 (td, *J*=7.6, 2.60 Hz, 2H).

NMM-IX (**14**)[7]

N-Methylmesoporphyrin IX dimethyl ester **13** (0.14 g, 0.23 mmol, 1.0 equiv) was dissolved in a THF/MeOH/H_2O mixture (15 mL, 1:1:1). LiOH·H2O (0.096 g, 2.3 mmol, 10.0 equiv) was added and the reaction mixture was stirred for 8 h at room temperature. The reaction mixture was filtered, and the volume of the filtrate was reduced under vacuum (5 mL). Citric acid (1N, 2 mL) was slowly added to this solution with stirring to precipitate the dark violet solid, which was filtered off and then washed with water (5 mL) to give **14** as a dark violet solid (the mixture **14a** and 1**4b**). Yield: 0.134 g (100%). Rf = 0.8 (CH_2Cl_2/MeOH 1:1). **14a**. rt = 1.67 min. LC-MS: m/z [M+H]+ = 581 Da, **14b**. rt = 1.68 min. LC-MS: m/z [M+H]+ = 581 Da.
^1H NMR (400 MHz, $CDCl_3$) *δ*: 11.86 - 12.93 (m, 2 H), 9.72 - 10.98 (m, 4 H), 3.40 - 4.83 (m, 19 H), 2.55 - 3.30 (m, 6 H), 1.70 - 1.91 (m, 4 H), 1.18 - 1.34 (m, 2 H)

Synthesis of biotinylated NMM derivatives

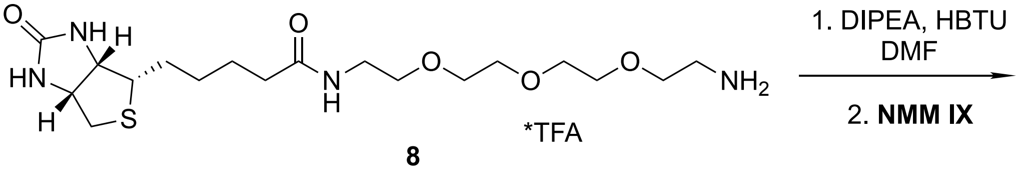

8

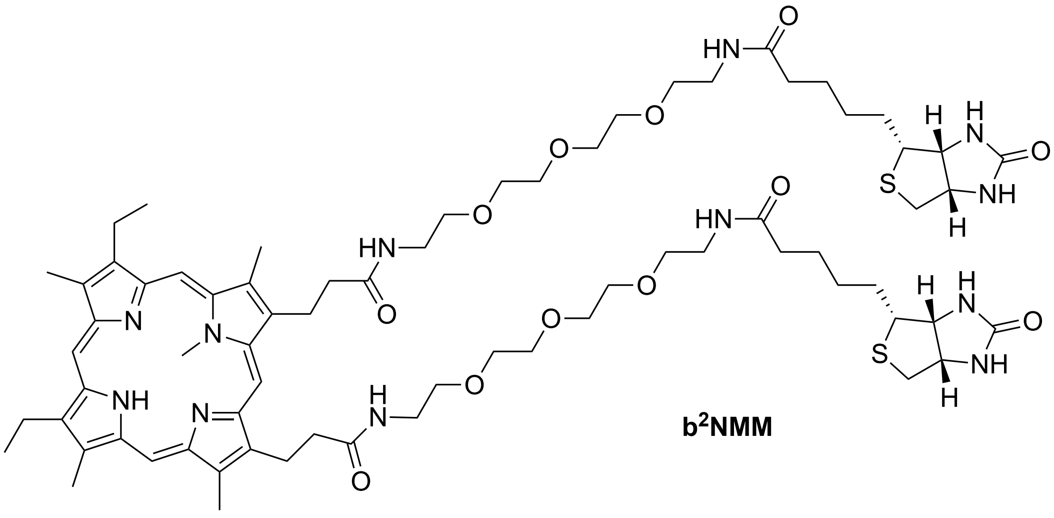

b²NMM

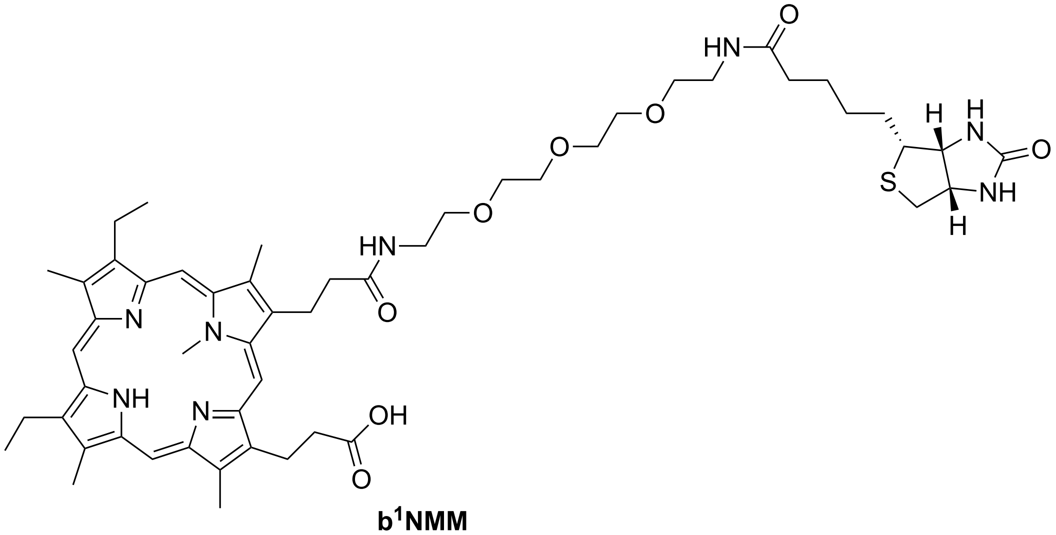

b¹NMM

Preparation of b²NMM and b¹NMM

HBTU (98%, 0.18 g, 9.00 mmol, 2.2 equiv) was added to a solution of NMM-IX **14** (0.123 g, 0.211 mmol, 1.0 equiv) in anhydrous DMF (5 mL), which was stirred for 2 h at room temperature. To this solution was added a solution of **9**·HCl (0.294 g, 0.561 mmol, 2.66 equiv.) and DIPEA (98%, 0.07 g, 0.09 mL, 0.528 mmol, 2.5 equiv) in anhydrous DMF (4 mL) and the solution mixture was stirred for 48 h at room temperature. After filtering, the filtrate was evaporated to dryness. The residual solid was dissolved in CH_2Cl_2 and the solution was washed with water, dried over anhydrous Na_2SO_4, and evaporated under reduced pressure to obtain a purple solid. The crude material was purified via preparative LC/MS with the following conditions: Column: YMC-Actus Pro C18, 20×250 mm, 12-μm particles; Mobile Phase A: 2:98 CH_3CN:H_2O with 0.1% formic acid; Mobile Phase B: 95:5 CH_3CN:H_2O with 0.1% formic acid; Gradient: 25-65% B over 15 minutes, then a 5-minutehold at 100% B; Flow: 20

mL/min. Fractions containing the desired products were combined and dried via centrifugal evaporation.
The yield of the **b²NMM** was 35.6 mg, and its estimated purity by LCMS analysis was 100%. Retention time=2.22 min; HRMS (ESI-MS) calcd for $C_{71}H_{105}N_{12}O_{12}S_2$ $[M+H]^+$ 1381.7411, found 1381.7338.
The yield of the **b¹NMM** was 8.7 mg, and its estimated purity by LCMS analysis was 100%. Retention time=2.31 min; HRMS (ESI-MS) calcd for $C_{53}H_{73}N_8O_9S$ $[M+H]^+$ 997.5222, found 997.5216.

Supplementary References

1 Lyons, S. M., Gudanis, D., Coyne, S. M., Gdaniec, Z. & Ivanov, P. Identification of functional tetramolecular RNA G-quadruplexes derived from transfer RNAs. *Nature Communications* **8**, 1127, doi:10.1038/s41467-017-01278-w (2017).

2 Morris, M. J. & Basu, S. An Unusually Stable G-Quadruplex within the 5′-UTR of the MT3 Matrix Metalloproteinase mRNA Represses Translation in Eukaryotic Cells. *Biochemistry* **48**, 5313-5319, doi:10.1021/bi900498z (2009).

3 Davila, J. *et al.* Cyto-mechanoresponsive Polyelectrolyte Multilayer Films. *Journal of the American Chemical Society* **134**, 83-86, doi:10.1021/ja208970b (2012).

4 Lebedeva, N. S., Yurina, E. S., Gubarev, Y. A., Kiselev, A. N. & Syrbu, S. A. Albumin aggregation promoted by protoporphyrin in vitro. *Mendeleev Communications* **30**, 211-213, doi:https://doi.org/10.1016/j.mencom.2020.03.027 (2020).

5 Rebouças, J. S. & James, B. R. A simple, catalytic H2-hydrogenation method for the synthesis of fine chemicals; hydrogenation of protoporphyrin IX dimethyl ester. *Tetrahedron Letters* **47**, 5119-5122, doi:https://doi.org/10.1016/j.tetlet.2006.05.083 (2006).

6 Honeybourne, C. L., Jackson, J. T., Simmonds, D. J. & Jones, O. T. G. A study of porphyrin analogues—III: Syntheses, enzyme interactions and self-aggregation of new models for types I, III and IX porphyrins. *Tetrahedron* **36**, 1833-1838, doi:https://doi.org/10.1016/0040-4020(80)80083-4 (1980).

7 El-Zaria, M. E., Ban, H. S. & Nakamura, H. Boron-Containing Protoporphyrin IX Derivatives and Their Modification for Boron Neutron Capture Therapy: Synthesis, Characterization, and Comparative In Vitro Toxicity Evaluation. *Chemistry – A European Journal* **16**, 1543-1552, doi:https://doi.org/10.1002/chem.200901532 (2010).
